# Overexpression of Tear Inflammatory Cytokines as Additional Finding in Keratoconus Patients and Their First Degree Family Members

**DOI:** 10.1155/2018/4285268

**Published:** 2018-09-02

**Authors:** Ioana Catalina Ionescu, Catalina Gabriela Corbu, Cristiana Tanase, Gabriela Ionita, Cristina Nicula, Valeria Coviltir, Vasile Potop, Mihaela Constantin, Elena Codrici, Simona Mihai, Ionela Daniela Popescu, Ana-Maria Enciu, Dana Dascalescu, Miruna Burcel, Radu Ciuluvica, Liliana-Mary Voinea

**Affiliations:** ^1^Clinical Hospital of Ophthalmologic Emergencies, Alexandru Lahovari 1 Square, Bucharest, Romania; ^2^Oftaclinic Ophthalmology Clinic, Bd. Marasesti 2B, Bucharest, Romania; ^3^Division of Ophthalmology, Faculty of Medicine, University of Medicine and Pharmacy “Carol Davila”, Bulevardul Eroii Sanitari 8, Sector 5, 050474 Bucharest, Romania; ^4^Biochemistry-Proteomics Department, Victor Babes National Institute of Pathology, Splaiul Independentei 99-101, Sector 5, 050096 Bucharest, Romania; ^5^Faculty of Medicine, Titu Maiorescu University, Strada Dâmbovnicului 22, Sector 4, 040441 Bucharest, Romania; ^6^Institute of Chemistry Physics “Ilie Murgulescu”, 202 Splaiul Independentei, 060021 Bucharest, Romania; ^7^Division of Ophthalmology, Faculty of Medicine, Iuliu Hatieganu University of Medicine and Pharmacy, Cluj-Napoca, Romania; ^8^Clinical Emergency Hospital Cluj-Napoca, Clinicilor Street 3-5, 400006 Cluj-Napoca, Romania; ^9^Cellular and Molecular Medicine Department, University of Medicine and Pharmacy “Carol Davila”, No. 8 B-dul Eroilor Sanitari, 050474 Bucharest, Romania; ^10^Faculty of Dental Medicine, University of Medicine and Pharmacy “Carol Davila”, Bulevardul Eroii Sanitari 8, 050474 Bucharest, Romania; ^11^Department of Ophthalmology, University Emergency Hospital, Splaiul Independent, ei 169, 050098 Bucharest, Romania

## Abstract

Keratoconus is a progressive corneal ectasia that may lead to severe visual impairment due to the irregular astigmatism caused by corneal thinning. In addition to its association with atopy, eye rubbing, or genetic component, late reports suggest the involvement of inflammation in the pathogenesis of the disease. Our aim was to determine the concentration of IL-4, IL-6, IL-10, RANTES, IFN gamma, and TNF alpha in the tear film of patients with keratoconus and their first degree family members. We analyzed forty-eight participants in an observational cross-sectional study. The diagnosis of keratoconus had to be confirmed in addition to a minimum of 47 D corneal refractive power by corneal topography readings provided by a Placido-based topography system and analysis of the pattern: irregular astigmatism with an asymmetric “bow-tie.” As for the other groups, the most important diagnosis criteria were a normal topographic pattern with a regular astigmatism. 17 keratoconus patients, 16 relatives, and 15 controls were recruited after clinical assessment as part of the research. The cytokine's mean values were similar in the keratoconus group and the relatives' samples but significantly higher compared to the controls. Important differences were found in IL-4 levels between keratoconus patients and relatives and between relatives and controls (mean difference of 302.42, *p* < 0.0016 and 219.16, *p* < 0.033, Tukey's HSD procedure). In the keratoconus group, using the CORR procedure, we found statistically strong correlations of IL-6 lacrimal concentrations with the disease stage (*r* = 0.56, *p* < 0.01), keratometry (*r* = 0.55, *p* < 0.02), pachymetry (*r* = −0.64, *p* < 0.048), and corneal hysteresis (*r* = −0.53, *p* < 0.02). Cytokine overexpression may be relevant for the inflammatory etiology of keratoconus. In conclusion, in the case of some first degree family members, the elevated tear biomarkers may represent a supplementary risk factor.

## 1. Introduction

Keratoconus is a corneal ectasia described as an asymmetric and progressive condition with significant consequences on the visual acuity and implicit on the patient's quality of life [1]. Keratoconus is characterized by progressive thinning of the corneal stroma leading to structural alteration of the tissue. The cone-shaped cornea induces irregular astigmatism. The corneal ectasia affects typically young adults at puberty and can progress until the 3rd–4th decade when the physiological corneal crosslinking is considered to be the stabilizing factor of the disease [2]. The exact etiology of keratoconus still remains unknown. Although most cases of keratoconus are sporadic, many studies have reported an important number of familial keratoconus inherited either through an autosomal dominant or recessive pattern. Recent researches describe the risk of first degree family members with positive family history of developing keratoconus [3, 4]. The LOX (collagen crosslinking enzyme lysyl oxidase) gene responsible for the crosslinking of collagen and elastin is considered a potentially dependable factor in the development of keratoconus, leading to a weakened corneal structure. Other genes such as CAST, DOCK9, IL1RN, SLC4A11, HGF, TGFBI, ZNF469, ZEB1, and VSX1 have been studied and involved in the possible genetic element of keratoconus [5, 6]. As for environmental factors, eye rubbing is one of the most important ones being closely connected to atopy. Subjects with allergic states have a higher risk of developing keratoconus [7].

Keratoconus was first described as a noninflammatory ectasia. On the contrary, multiple recent studies published evidence that highlight a possible inflammatory etiology [8–11] marked through the presence of proinflammatory cytokines such as IL-6, IL-1 beta, IFN gamma, and TNF alpha in the tear film of keratoconus patients.

A novelty in the pathogenesis of keratoconus is the imbalance of inflammatory and immune responses marked by the T-helper cells (Th). Th cells are divided into 5 distinct subtypes, 2 of which are relevant in our study: Th1 and Th2. While the cells that differentiate into Th1 promote the immune cellular response by stimulating the production of cytotoxic T cells and increasing the macrophage's activity, Th2 cells contribute to the development of allergic states, inducing the proliferation of eosinophiles and mastocytes [12]. Th1 cells produce IL-2, IL-3, IFN gamma, and TNF alpha and beta. The effector Th2 cells synthesize IL-4, IL-5, IL-6, IL-10, and IL-13 [13, 14]. IL-1, made up of 2 proteins IL-1 alpha and IL-1 beta, has as cell sources the macrophages and monocytes, and their targets are the T cells, fibroblasts, and epithelial cells, resulting in induction of proinflammatory proteins. IL-4 derives from Th2 cells and targets the T and B cells, stimulating the production of B cells and upregulating the expression of class II MHC on B cells [15]. As for IL-6, it seems to affect the pathogenesis of many autoimmune and inflammatory diseases. In vitro studies revealed that IL-6 transsignaling increases chemokine activation of CXCL5, CXCL6, and CCL8 [16]. The Th2-derived factor, IL-10, downregulates the surface expression of class II MHC molecules and inhibits the action on certain cytokines like IL-1 beta, IL-6, IL-10, and TNF alpha [17, 18]. As the product of fully differentiated Th1 cells, IFN gamma controls a broad range of biological functions. IFN gamma modulates proapoptotic activity and is capable of promoting apoptosis by enhancing the surface expression of the TNF alpha receptor [19]. In addition, IFN gamma upregulates some lysosomal proteases such as cathepsins B and L [20]. The tumor necrosis factor is produced by activated macrophages and CD4+ lymphocytes with a complete signaling pathway. TNF alpha is also involved in the induction of cell apoptosis [21].

## 2. Methods

The present study was performed in adherence to the Declaration of Helsinki, and prior to their enrollment in the study, we obtained written informed consent from all participants. The study followed the institutional ethics guidelines and was approved by the ethics committee of the University of Medicine and Pharmacy “Carol Davila,” Bucharest. All 48 subjects were recruited from Oftaclinic Ophthalmology Clinic, Bucharest, Romania, from January 2016 to July 2017.

### 2.1. Patients

The prospective study included forty-eight patients divided into three groups. We initially recruited 17 pairs of keratoconus relatives. But given the fact that one family member had borderline changes on the topography readings, we decided to exclude the subject. Thus, the study consists of 17 patients with keratoconus, 16 first degree family members of the patients, and 15 control subjects. The inclusion criterion for the keratoconus group was the positive diagnosis of keratoconus assessed by corneal topography, pachymetry, corneal biomechanics, and slit lamp examination with the following clinical signs depending on the disease stage: paracentral stromal thinning, “oil droplet” reflex, Vogt striae, Fleischer ring, and Munson sign. As for the groups of first degree family members and controls, the criteria were represented by a normal biomicroscopic examination, normal corneal topography readings, and normal biomechanical measurement. The exclusion criteria for all three polls were all subjects with ocular or systemic allergy, the use of contact lenses, history of ocular surgery (corneal collagen crosslinking, pterygium, cataract surgery, and refractive surgery), current systemic or localized inflammatory, autoimmune conditions, and dry eye disease.

### 2.2. Procedures

All eyes underwent the following ophthalmic examinations:

- Complete personal and family history taking including the use of any type of anti-inflammatory, antiallergic ocular, or systemic medications.
- Clinical examination: best corrected visual acuity and slit lamp examination. (Subjects with a BUT over 10 seconds and Schirmer's test of minimum of 10 mm were included in the study.)
- Paraclinical examinations: corneal topography (Topcon), Ocular Response Analyzer (ORA, Reichert, Depew, NJ), pachymetry (or central corneal thickness (CCT)), and topographic indices (maximum keratometry reading (Kmax) and the keratoconus prediction index (KPI)). The stage of keratoconus was graded with respect to the Amsler-Krumeich classification as stage I (mean *K* < 48 D), stage II (48–53 D, CCT > 400 *μ*m), stage III (53–55 D, CCT = 300–400 *μ*m), and stage IV (>55 D, CCT < 300 *μ*m). Regarding corneal biomechanics, we evaluated certain in vivo parameters using ORA such as the corneal hysteresis (CH), corneal resistance factor (CRF), and the keratoconus match probability (KMP).

At the time of tear collection, no participant gave a history of local or systemic allergy or inflammation. In the cases of unilateral keratoconus, we chose to collect the fluid from the diseased eye. Furthermore, some keratoconus patients had already undergone one eye corneal crosslinking and thus included in the study the unaffected eye. The challenge of the study was to respect all inclusion and exclusion criteria in order to have results as accurate as possible. Tear collection was performed carefully without topical anesthesia, in a low illuminated room. 50 *μ*L capillary tubes were used to collect a minimum of 15 *μ*L of tear volume from the inferior conjunctival cul-de-sac (avoiding reflex tearing) by capillary attraction and then transferred into Eppendorf tubes. For every tear sample, we used new microcapillaries and Eppendorf microtubes. Following the collection, the tear samples were stored at −80°C within 1 hour without centrifugation until analysis.

The MILLIPLEX MAP Cytokine/Chemokine Magnetic Bead Panel Kit 7-plex panel comprising IL-1 beta, IFN gamma, IL-10, IL-4, IL-6, RANTES, and TNF alpha (Merck Millipore, Billerica, MA, USA) was assessed according to the manufacturer's instructions [22]. The standard concentrations were prepared, making serial dilutions, from 3.2 to 10,000 pg/mL, and the assay buffer was considered as background (standard 0 pg/mL). Briefly, the samples (tears) were incubated with the bead sets, buffer, and cytokine standards, Quality Controls 1 and 2, provided within the kit in a 96-well plate at 4°C overnight. A volume of 25 *μ*L of standard/controls/samples was added in the corresponding wells, followed by 25 *μ*L of assay buffer and 25 *μ*L of bead mixture. All further incubations, including biotinylated detection antibody addition, followed by incubation with streptavidin-phycoerythrin, were achieved at room temperature, in the dark, with shaking at 500 rpm. The required wash steps were performed using a magnetic plate washer. Multiplex data acquisition and analysis were performed using a Luminex 200 platform (Luminex Corp., Austin, TX, USA) and xPONENT software version 3.1. Each individual microsphere was recognized by its own “bead signature” and quantified afterwards, based on the fluorescent reporter signals. The ranges for each studied analyte in Quality Controls 1 and 2 were also provided within the card insert of the kit, and the Quality Control ranges were generated with overnight assay format using serum matrix provided in the MILLIPLEX kit. Duplicate samples were used for all specimens, and the calibration curves were generated with a 5-parameter logistic fit. The curves were adequately adjusted for each analyte in order to optimally fit the expected ranges. Regarding the assay sensitivities, minimum detectable concentrations, measured in pg/mL, were calculated using the xPONENT 3.1 software, also provided in the kit for each individual analyte. The lowest detectable concentration fell within the 0.4–0.8 pg/mL range. The data was presented as the mean SD of duplicate, and the two-tailed *p* values of less than 0.05 were considered to indicate significant differences using Student's *t*-test.

### 2.3. Statistical Analysis

Patients' data were collected and introduced into an OpenOffice PC, version 4.1.1© 2014. The statistical analysis was completed with the following programs: SAS University Edition version 9.3 and R version 3.4.0.

#### 2.3.1. Descriptive Statistics

For the categorical variables, we determined the absolute and relative frequency. We used the boxplot for the graphical representations.

#### 2.3.2. Interferential Statistics

The comparison between the continuous variables of the 3 polls was made as follows: if the distribution of the variables on the 3 batches could be approximated by a Gaussian distribution, an ANOVA test was initially used. If the ANOVA test revealed statistically significant differences, we also performed a post hoc post-ANOVA procedure called Tukey's HSD test. If the distribution of the variables could not be approximated by a normal distribution, a Kruskal-Wallis test was performed followed by a Dunn test (post hoc post-Kruskal-Wallis procedure) in the case of a statistically significant result. The correlations between the variables were investigated by determining the *r* Pearson correlation index (if the distribution of the variables could be approximated with a normal one) or *ρ* Spearman correlation index (if the distributions could not be determined by a Gaussian distribution). Tests with a *p* value ≤ 0.05 were considered statistically significant.

## 3. Results

A total of 48 patients were included in the present study: 17 eyes of 17 patients with keratoconus (64.71% males and 35.29% females), 16 eyes of 16 keratoconus first degree relatives (56.25% males and 43.75% females), and 15 controls of 15 normal eyes (40% males, 60% females). In the set of keratoconus, the age ranged from 13 to 59 with a mean of 23.35 (±11.80; *p* value = 0.0006) while the relatives' group had a mean age of 18.81 (±6.13; *p* value = 0.7645) ranging from 9 to 30 and the controls had a mean age of 28.66 (±3.03; *p* value = 0.9529) with a range between 23 and 34. Minimal age-related differences between the 3 groups are present, but all subjects are included in the category of young adults.

According to the Amsler-Krumeich classification, 3 (18%) of the keratoconus patients were graded as stage I, 5 (29%) as stage II, 4 (24%) as stage III, and 5 (29%) as stage IV.

**Figure 1:**
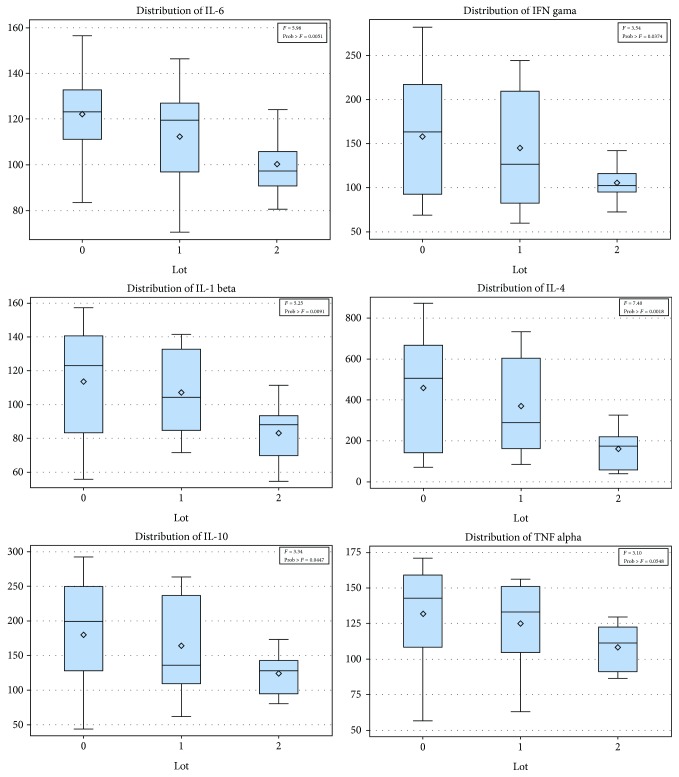
Distribution of cytokines in the tear fluid of subjects. 0 = keratoconus group; 1 = family members; 2 = control group. It is noticeable that the tear cytokine concentration is highest in the keratoconus group, followed by the family members' group, and lowest in the control group.

To investigate the role of inflammation in keratoconus and to analyze whether there is also an inflammatory component in the first degree relatives of the keratoconus patients, we performed an observational cross-section study. All cytokines tested by the xMAP assay were detectable in tears except for 1 patient in the case of IL-1 beta and 2 patients for IL-4. Their mean values and distributions are shown in Table 1 and Figure 1.

**Figure 2:**
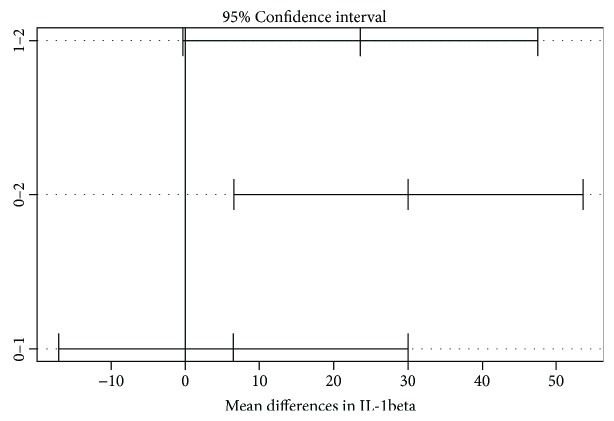
Mean differences between groups shown with 95% confidence interval in IL-1 beta. 0: keratoconus group; 1: family members' group; 2: control group. There were significant differences between the groups 1 and 2 as well as between 0 and 2.

**Figure 3:**
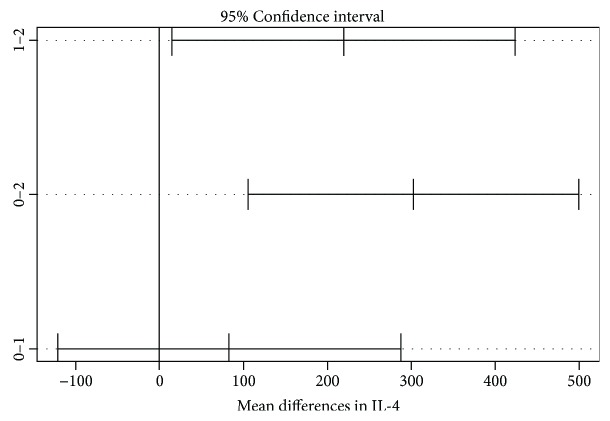
Mean differences between groups shown with 95% confidence interval in IL-4. 0: keratoconus group; 1: family member group; 2: control group. There were significant differences between groups 1 and 2 as well as between 0 and 2.

The distribution of all variables above (cytokines) except for RANTES was normal in all groups (similar to the Gaussian distribution). In order to investigate whether the mean differences between the 3 groups have statistical significance, we used the ANOVA test and observed that the increase in almost all cytokines is statistically significant with the exception of RANTES where there were no important differences between the 3 polls. While the ANOVA test showed in all variables statistical significance (*p* < 0.05), after Tukey's honest significant difference test, the results suffered a change. The means of IFN gamma, IL-10, IL-1 beta, IL-4, IL-6, and TNF alpha had a significant difference between the keratoconus patients and the normal subjects. IL-1 beta and IL-4 presented the most notable significant differences between the keratoconus and control groups as well as between the relatives and normal subjects. There were no statistically significant differences between the keratoconus patients and their relatives, as seen in Tables 2 and 3 and as ilustrated in Figures 2 and 3.

Further on, we focused our study on the keratoconus and control groups and carried out some correlations between cytokines and important parameters of the disease. We carried out the correlation between tear cytokines level and the severity of the disease in the keratoconus group. Weak and medium, yet positive, correlations were found but not all showed a statistical significance. We observed medium positive correlation with statistical significance except for IL-10 and TNF alpha, meaning that a progression in the disease's stage is accompanied by a higher value of inflammatory tear biomarkers.

The tear inflammatory biomarker level was directly correlated with the keratometry reading. The results, as shown in Table 4, evidence a positive, medium, and statistically significant correlation in the case of IL-6. As for CCT in keratoconus patients, we found negative, strong, and statistically significant correlations. Analyzing Table 4, we can state that IL-6 presented the most significant correlations in relation to severity, keratometry, and pachymetry (*r* = +0.5621, *p* < 0.0188; *r* = +0.55, *p* = 0.02; and *r* = −0.6489, *p* < 0.0048, resp.). A positive and direct correlation in the case of keratometry means that an increase in the keratometry readings is accompanied by an overexpression of tear cytokines. On the other hand, a negative correlation concerning pachymetry suggests the fact that the lower the central corneal thickness, the higher the cytokine concentration in the tears of keratoconus patients.

Moreover, for the study of corneal biomechanics in keratoconus, we examined the correlation between CH and CRF and cytokines and discovered negative strong correlations between the variables, suggestive of the fact that an increase in the level of tear cytokines is accompanied by a decrease in CH and CRF as reflected in Table 2, most notable in the case of IL-10 (*r* = −0.87 and *p* < 0.0001 for CH and *r* = −0.87 and *p* < 0.0001 for CRF).

## 4. Discussion

Extensive studies on the etiology of keratoconus have been made, but it still remains unclear. The role of cytokines, proteases, and oxidative stress is a central debate among the researchers, since it is hypothesized that inflammation mediators in the tear film of keratoconus patients could be one of the causes for the tissue degradation in the diseased cornea and for the progression of the ectasia.

Many studies relate the corneal thinning to a microenvironmental imbalance between the increased levels of proteolytic enzymes and the decreased levels of their inhibitors [23].

Galvis et al. concluded in a complete and structured review on inflammation in keratoconus the existence of a vicious circle between inflammatory cytokines, proteases on the one hand and their inhibitors on the other, and an overexpression of oxidative stress, leading to increased apoptosis [24].

To our knowledge, this study has examined for the first time in Romania the concentration of inflammatory cytokines in the tears of keratoconus patients and their first degree family members. Since the genetic factor is a demonstrated fact, we took into account the possibility of local inflammatory changes in the patients' relatives. We found increased expression of cytokines in both groups (keratoconus patients and their relatives) compared to the controls.

Therefore, we reviewed the most relevant studies in the literature concerning the inflammatory pathway in keratoconus in order to compare our results. Only a few studies were performed by an immune bead-based multiplex kit, whereas many used the standard ELISA test with a lower sensitivity than the multiplex assay. Our results indicated elevated expression of IL-1b, IL-4, IL-6, IL-10, IFN gamma, and TNF alpha in the tears of keratoconic eyes as well as in the second group (relatives) compared to the control subjects. The IL-1 family of cytokines has strong proinflammatory actions and is responsible for the activation of collagenases and metalloproteinases, as well as for the overexpression of IL-6 [25, 26].

In a study conducted by Pouliquen et al., keratoconic corneas presented increased IL-1 receptor expression compared to normal corneas [27]. According to Wilson et al.'s experiment, IL-1b could induce cell apoptosis in the corneal stroma, altering the normal architecture of the tissue in keratoconus patients [28]. In our study, we found increased levels of IL-1b in both the keratoconus and the relatives' groups compared to the normal subjects. This result was in accordance with Sorkhabi et al. but in contrast with Jun et al. where the IL-1b tear level remained unchanged in keratoconus patients [9, 29].

Below is an overview of studies on inflammatory mediators in the tear fluid in keratoconus measured by different cytokine antibody arrays. In 2010, Pannebaker et al. reported no statistically significant difference in cytokine levels between keratoconus eyes and normal participants. On the other hand, they revealed a significant increase in matrix metalloproteinase-one (MMP-1) in the keratoconus group [30]. Cytokine measurements in the study conducted by Jun et al. showed increased IL-6 and decreased TNF alpha, IFN gamma, IL-4, and CCL5 in keratoconus compared to control tears [29]. Our findings confirm an increased IL-6 tear level, while the other biomarker levels are in contrast with our results. The possible reasons for these differences could be the use of different immune bead-based multiplex systems and the significant differences in the patients' mean age between the studies. While Jun et al. recruited patients with a mean age of 38 ± 10 for keratoconus subjects, in our study, we had a mean age of 23.35 with a standard deviation of 11.08. Although the scopes of the studies were small, we could raise the question whether age influences the immune response and implicit the disease progression, since there is an interplay between these factors. The hypothesis that in older keratoconus patients the inflammatory interactions are very low could explain the differences between the conflicting findings. Balasubramanian et al. published in 2012 a series of results suggesting increased expressions of IL-4, IL-5, TNF alpha, IL-10, and IL-6 in the tears of keratoconus patients, hence classifying keratoconus as an inflammatory disease. These findings were similar to ours, as well as the positive correlation of the cytokines to keratometry readings [31].

Using the conventional ELISA test, Lema et al. observed increased levels of IL-6 and TNF alpha in keratoconic eyes, which are accordant with our study, while Sorkhabe et al. measured a decreased level of IL-10 compared to normal subjects, a situation that contradicts our results [8, 9].

Kolozsvari et al. reflected on the correlation between the severity of keratoconus and the tear cytokines and proved a positive correlation between CCL5 (RANTES), respectively, IL-6 and a keratometric index, indicating that the higher the local inflammation, the more important the severity of the disease [10]. This observation was also found in our present study, showing medium positive correlation between IL-1b, IL-4, and IL-6 and the severity of the corneal ectasia.

This is the first study in Romania to correlate tear biomarkers with corneal biomechanics. Regarding the literature, there are many studies that reveal the significantly reduced values of corneal hysteresis and corneal resistance factor in keratoconic eyes compared to healthy eyes as well as their role in monitoring the disease progression. Our experiment is consistent with the published results, stating once again that keratoconus patients have altered biomechanical properties [32–35]. Going further, we found that the inflammatory cytokines in the keratoconus group are negatively and statistically significantly correlated to the two important parameters, CH and CRF.

To the best of our knowledge, this is the first study in Romania evaluating the inflammatory state in keratoconus' first degree family members. Our results, which could have more global considerations, show an overexpression in the level of several cytokines compared to controls. Although these measurements cannot predict whether the relatives will develop or not the disease, they could be taken into account for a more extensive screening.

Our study has limitations such as a small number of participants and the lack of cytokine serum measurement. We have based our study on the study conducted by Jun et al. that found no significant differences between keratoconus patients and normal individuals regarding the serum cytokine concentrations, suggesting that keratoconus is not associated with systemic inflammation [29]. However, despite its limitations, this present report could be regarded as a pilot study that needs further intensive research.

## 5. Conclusions

In summary, this study reveals alterations in cytokine concentrations in the tears of patients with keratoconus and their first degree family members, supporting the hypothesis of inflammatory signaling in the pathway of the disease. In addition, we raise the question of diagnostic accuracy concerning the tear inflammatory biomarkers as well as the matter of diagnostic ability of the cytokine levels as an additive parameter to corneal imaging tests, a potential focus for future research studies. The data regarding the relatives cannot yet confirm the possible risk factor in developing keratoconus; hence, prospective studies over years are required to investigate and closely monitor first degree family members of keratoconus patients and to confirm the elevated tear mediators in a larger population.

## Figures and Tables

**Table 1:** Tear-fluid cytokine mean concentrations measured in pg/mL in keratoconus, first degree family members, and controls.

Cytokines (pg/mL)	Keratoconus	First degree family members	Controls	*p* value (test)
IFN gamma	157.75 (±69.68)	144.59 (±63.61)	106.07 (±19.93)	0.0374 (ANOVA)
IL-10	181.61 (±78.55)	163.60 (70.97)	123.56 (±30.96)	0.0447 (ANOVA)
IL-1 beta	113.52 (±34.86)	107.06 (±24.71)	83.45 (±18.07)	0.0091 (ANOVA)
IL-4	461.67 (±283.20)	378.37 (±242.48)	159.21 (±99.72)	0.0018 (ANOVA)
IL-6	122.32 (±18.63)	112.41 (±20.38)	100.31 (±14.13)	0.0051 (ANOVA)
RANTES	185.37 (±38.73)	169.61 (±37.95)	174.14 (±65.67)	0.3061 (Kruskal-Wallis)
TNF alpha	131.80 (±34.29)	125.33 (±28.08)	108.07 (±15.84)	0.05 (ANOVA)

**Table 2:** Mean differences between groups for IL-1 beta measured with Tukey's honest test.

IL-1 beta	Mean difference	Tukey-adjusted *p* value	95% confidence interval
Keratoconus versus relatives	6.46	0.7842	−17.09 to 30.02
Keratoconus versus controls	30.07	0.0094	6.51 to 53.63
Relatives versus controls	23.60	0.0539	−0.33 to 47.54

**Table 3:** Mean differences between groups for IL-4 measured with Tukey's honest test.

IL-4	Mean difference	Tukey-adjusted *p* value	95% confidence interval
Keratoconus versus relatives	83.29	0.5866	−121.25 to 287.85
Keratoconus versus controls	302.45	0.0016	105.34 to 499.57
Relatives versus controls	219.16	0.0333	14.60 to 423.71

**Table 4:** Correlations between the concentrations of inflammatory mediators and paraclinical parameters in keratoconus patients.

	Pearson/Spearman correlation coefficient						
	*p* value						
	Number of observations						
Cytokine	IFN gamma	IL-10	IL-1 beta	IL-4	IL-6	RANTES	TNF alpha
Disease stage	0.41420 0.0983 17	0.2867 0.2644 17	0.48542 0.0566 16	0.4971 0.0594 15	0.5621 0.0188 17	0.49538 0.0432 17	0.15387 0.5554 17

Kmax	0.25383 0.3256 17	0.17647 0.4981 17	0.3529 0.1800 16	0.3178 0.2483 15	0.55637 0.0204 17	0.37255 0.1408 17	0.16912 0.5164 17

CCT	−0.54897 0.0225 17	−0.4543 0.0669 17	−0.62387 0.0098 16	−0.6076 0.0163 15	−0.6489 0.0048 17	−0.65120 0.0046 17	−0.37691 0.1359 17

CH	−0.86258 <0.0001 17	−0.8767 <0.0001 17	−0.81825 0.0001 16	−0.8328 0.0001 15	−0.5395 0.0254 17	−0.50766 0.0375 17	−0.86205 <0.0001 17

CRF	−0.82852 <0.0001 17	−0.8739 <0.0001 17	−0.79039 0.0003 16	−0.8089 0.0003 15	−0.3630 0.1521 17	−0.41664 0.0962 17	−0.84797 <0.0001 17

In the case of 1 subject, IL-1 beta was not detectable in the tear film, the same as in the case of 2 subjects for IL-4, hence the missing observations. Kmax: maximal keratometry reading measured by the corneal topography; CCT: central corneal thickness measured by ultrasonic pachymeter or by OCT; CH: corneal hysteresis; CRF: corneal resistance factor measured with the Ocular Response Analyzer.

## Data Availability

The data (among Excel, SAS University Edition version 9.3 and R - version 3.4.0 were used for data analysis) used to support the findings of this study are available from the corresponding author upon request.
